# Cerebrospinal fluid biomarkers for predicting development of multiple sclerosis in acute optic neuritis: a population-based prospective cohort study

**DOI:** 10.1186/s12974-019-1440-5

**Published:** 2019-03-11

**Authors:** M. N. Olesen, K. Soelberg, B. Debrabant, A. C. Nilsson, S. T. Lillevang, J. Grauslund, I. Brandslund, J. S. Madsen, F. Paul, T. J. Smith, S. Jarius, N. Asgari

**Affiliations:** 1 Department of Neurology, Slagelse Hospital, Slagelse, Denmark; 2 Department of Internal Medicine, Slagelse Hospital, Slagelse, Denmark; 3 Epidemiology, Biostatistics and Biodemography, Department of Public Health, University of Southern Denmark, Odense, Denmark; 4 Department of Clinical Immunology, Odense University Hospital, Odense, Denmark; 5 Department of Ophthalmology, Odense University Hospital, Odense, Denmark; 6 Department of Clinical Research, University of Southern Denmark, Odense, Denmark; 7 Department of Clinical Immunology and Biochemistry, Lillebælt Hospital, Vejle, Denmark; 8 Institute of Regional Health Research, University of Southern Denmark, Odense, Denmark; 9 Clinical and Experimental Multiple Sclerosis Research Center and NeuroCure Clinical Research Center, Department of Neurology, Charité - Universitätsmedizin Berlin, Berlin, Germany; 10 Experimental and Clinical Research Center, Max Delbrueck Center for Molecular Medicine and Charité – Universitätsmedizin Berlin, Berlin, Germany; 11 Departments of Ophthalmology and Visual Sciences and Internal Medicine, University of Michigan Medical School, Ann Arbor, MI USA; 12 Molecular Neuroimmunology Group, Department of Neurology, University Hospital Heidelberg, Heidelberg, Germany; 13 Institutes of Regional Health Research and Molecular Medicine, University of Southern Denmark, Winsloewsvej 25.2, 5000 Odense C, Denmark; 14 Odense Patient data Explorative Network (OPEN), Odense University Hospital, Odense, Denmark

**Keywords:** Immunology, Optic neuritis, Multiple sclerosis, Cerebrospinal fluid, Biomarkers, Inflammation, Neurodegeneration

## Abstract

**Background:**

Long-term outcome in multiple sclerosis (MS) depends on early treatment. In patients with acute optic neuritis (ON), an early inflammatory event, we investigated markers in cerebrospinal fluid (CSF), which may predict a diagnosis of MS.

**Methods:**

Forty patients with acute ON were recruited in a prospective population-based cohort with median 29 months (range 19–41) of follow-up. Paired CSF and serum samples were taken within 14 days (range 2–38), prior to treatment. Prospectively, 16/40 patients were by a uniform algorithm diagnosed with MS (MS-ON) and 24 patients continued to manifest isolated ON (ION) during follow-up. Levels of cytokines and neurofilament light chain (NF-L) were measured at the onset of acute ON and compared to healthy controls (HC). Significance levels were corrected for multiple comparisons (“q”). The predictive value of biomarkers was determined with multivariable prediction models using nomograms.

**Results:**

CSF TNF-α, IL-10, and CXCL13 levels were increased in MS-ON compared to those in ION patients (*q* = 0.021, 0.004, and 0.0006, respectively). MS-ON patients had increased CSF pleocytosis, IgG indices, and oligoclonal bands (OCBs) compared to ION (*q =* 0.0007, *q =* 0.0058, and *q =* 0.0021, respectively). CSF levels of IL-10, TNF-a, IL-17A, and CXCL13 in MS-ON patients correlated with leukocyte counts (*r* > 0.69 and *p* < 0.002) and IgG index (*r* > 0.55, *p* < 0.037). CSF NF-L levels were increased in ON patients compared to those in HC (*q =* 0.0077). In MS-ON, a progressive increase in NF-L levels was observed at 7 to 14 days after disease onset (*r* = 0.73, *p* < 0.0065). Receiver-operating characteristic (ROC) curves for two multivariable prediction models were generated, with IL-10, CXCL13, and NF-L in one (“candidate”) and IgG index, OCB, and leukocytes in another (“routine”). Area under the curve was 0.89 [95% CI 0.77–1] and 0.86 [0.74–0.98], respectively. Predictions of the risk of MS diagnosis were illustrated by two nomograms.

**Conclusions:**

CSF TNF-α, IL-10, CXCL13, and NF-L levels were associated with the development of MS, suggesting that the inflammatory and neurodegenerative processes occurred early. Based on subsequent diagnosis, we observed a high predictive value of routine and candidate biomarkers in CSF for the development of MS in acute ON. The nomogram predictions may be useful in the diagnostic work-up of MS.

**Electronic supplementary material:**

The online version of this article (10.1186/s12974-019-1440-5) contains supplementary material, which is available to authorized users.

## Introduction

Optic neuritis (ON) is an inflammatory optic neuropathy, which may occur as an early manifestation of multiple sclerosis (MS) [1]. ON is believed to be immune-mediated and results from the destruction of the myelin sheath and nerve fibers, resulting in impaired vision or blindness [2]. Antibodies with specificity for myelin oligodendrocyte glycoprotein (MOG) are implicated in ON, as well as in ON associated with neuromyelitis optica spectrum disorder (NMOSD) in cases where immunoglobulin (Ig) G antibodies against the water channel aquaporin-4 are undetectable [3–6]. The predilection for the optic nerve in MS may be explained by regional differences in blood-brain barrier (BBB) permeability. Significant risk factors for the transition of ON to MS routinely used in clinical practice are the presence of brain lesions on MRI and cerebrospinal fluid (CSF)-restricted oligoclonal bands (OCBs). These represent intrathecally produced IgG of unknown specificity [7, 8]. Apart from OCBs, alterations of the levels of interleukin (IL)-10 produced by naïve and/or regulatory B cells and the chemokine, CXCL13, which is a key regulator of B cell recruitment, have been reported in MS patients [9–11]. These findings suggest that B cells play a role in the pathogenesis of MS, although their importance in MS development has not yet been determined.

Neurofilament light chain (NF-L) is a major structural component of neurons. Increased levels of NF-L in CSF have been suggested to be a marker of axonal damage in MS [12]. The implications of early changes of NF-L levels in acute ON and its potential predictive value for the subsequent conversion to MS are unexplored. Furthermore, the relation between intrathecal inflammation and neurodegeneration in acute ON is incompletely understood. We propose that markers of inflammation and of neurodegeneration (a) may differ between patients with MS-related ON and patients with ON unrelated to MS and (b) may predict development of MS in patients with acute ON.

## Materials and methods

### Study design and patients

Patients with acute ON originated from a population-based prospective study with clinical follow-up performed 2014–2016 in the Region of Southern Denmark [13, 14]. The diagnosis of ON was obtained by independent neurological and ophthalmological examination as previously described based on a uniform algorithm [13, 14]. The neurologist and ophthalmologist were masked to results from autoantibody and cytokine/chemokine analysis, and the laboratory analyses were performed blinded to the clinical status of patients [14]. Patients were excluded if they had been diagnosed previously with MS or NMOSD. AQP4-IgG was measured with a recombinant immunofluorescence assay using HEK293 cells transfected with recombinant human full-length AQP4 gene and re-evaluated by means of an in-house cell-based assay [14]. MOG-IgG was determined by two cell-based assays employing fixed and live HEK293 cells, respectively, transfected with full-length human DNA [3, 6]. MS was diagnosed according to the 2010 MS criteria [8]. Forty patients presenting with acute ON prior to treatment and a median follow-up time after onset of 29 months (range 19–41) were included in the study. We tested and found no indication of immunological differences between males and females (not shown).

CSF samples were collected from 12 age-matched subjects assessed because of sudden transient headache without trauma or infection and where all subsequent investigations were normal. These individuals were considered healthy controls (HC) [15].

### Sample storage and handling

Venous blood and CSF from 40 patients were collected within 38 days of ON onset (median, 14 days; range 2–38). For IgG and albumin ratios, EDTA plasma was prepared and analyzed directly. CSF was sampled and stored according to international research standards [1]. Briefly, for quantification of IL-1β, IL-6, IL-10, IL-17A, tumor necrosis factor (TNF)-α, and TNF-related apoptosis-inducing ligand (TRAIL), CSF was collected, centrifuged immediately, cooled to 4 °C for 2 h, and stored at − 20 °C for up to 1 month and then at − 80 °C until analysis. Serum was isolated following clot formation at ambient temperature and frozen as described for CSF.

Interferon (IFN)-α, IFN-β, and CXCL13 concentrations were determined on an aliquot of the CSF and serum that had been frozen at − 80 °C immediately following centrifugation. Samples were thawed once for aliquoting prior to analysis, except for CXCL13 (one additional thawing cycle).

### Immunochemical and cytological parameters

Routine diagnostic analyses were carried out at the Department of Biochemistry and Immunology, Vejle Hospital, being accredited by Danish Accreditation Fund (DANAK) according to the ISO 15189 standard that specifies requirements for quality and competence in medical laboratories. IgG and albumin were measured on a Cobas 8000C instrument (Roche, Basel, Switzerland), oligoclonal bands (OCBs) on a Hydrasys instrument (Sebia, Surrey, UK), and leukocytes on a Sysmex XN-9000 instrument (Sysmex Europe, Norderstadt, Germany).

### Immunoassay measurements

IL-1β, IL-6, IL-10, IL-17A, TNF-α, TRAIL, and NF-L were measured using Simoa HD-1 digital enzyme-linked immunosorbent assay (ELISA) (Quanterix, Lexington, MA, USA).

CXCL13 and INF-α/β concentrations were determined using ELISAs (Euroimmun, Lübeck, Germany and PBL Assay Science, NJ, USA, respectively) in the Department of Clinical Immunology, Odense University Hospital, also accredited according to the ISO 15189 standard. Lower cutoff sensitivity of the CXCL13 assay was set to 10 pg/ml, according to the manufacturer’s instructions [16].

### Statistical analysis

#### Association analysis

Prism 7 (GraphPad Software, La Jolla, CA) was used for generating comparisons and correlation analyses with a significance level of 5%. Fisher’s exact test was used to test for differences regarding frequency distributions (sex and OCBs). Continuous variable data was analyzed using the nonparametric Kruskal-Wallis test. Results described in the text are given as “median [25^th^–75^th^ percentiles]” unless otherwise clearly stated. CSF-to-serum ratio of cytokines was normalized to albumin ratio and similarly compared by the Kruskal-Wallis test. To account for multiple testing, these analyses were followed by false discovery rate adjustment (alpha = 0.05) of *p* values, and corresponding *q* values were reported unless clearly stated [17]. Spearman correlations were estimated and tested for being identical to 0 without further *p* value adjustment. This approach for reducing false positives was chosen due to the explorative nature of the study.

#### Predictive modeling

R version 3.3 (R Core Team at R Foundation for Statistical Computing, Vienna, Austria) was used for extended analyses of the conversion of isolated optic neuritis (ION) to multiple sclerosis in relation to optic neuritis (MS-ON). Here, logistic regression modeling was performed, including receiver-operating characteristic (ROC) area under curve (AUC) calculations. For uni- and multivariate model assessment, we reported on likelihood ratios, overall *p* values, and McFadden’s adjusted *R*^2^, and for multivariate models also the Hosmer-Lemeshow’s goodness-of-fit test is reported. Since prediction performance measures such as AUCs are overly optimistic when calculated for the same sample population as used for model fitting, we also applied a bootstrap optimism correction based on 500 bootstrap samples. This is an internal validation method for assessing the optimism bias of prediction models when no validation cohort is available, i.e., when a model is fitted and tested on the same dataset. The approach calculates AUC_apparent_ − Optimism = AUC_corrected_, and is described in detail by Steyerberg [18]. When applying this approach, we did however not account for potential preselection of variables before inclusion in our multivariable prediction models, in which case the actual optimism will likely be larger than estimated. Nomograms as graphical output representations of the logistic regression models were made using R, and calibration plots showing observed versus expected proportions were used to assess calibration of the prediction models.

## Results

### Cohort characteristics

All 40 patients with acute ON were Caucasians with a female to male ratio of 2:1 and a median age of 36 years (range 16–66 years). A total of 12 patients (30%) were diagnosed with MS (MS-ON) at the acute stage of ON (< 2 months), and four additional patients developed MS within the first year of follow-up (giving a total of 16 patients, 40%, with MS-ON) [14]. The remaining 24 patients had a final diagnosis of isolated ON (ION). None of the patients had detectable AQP4-IgG according to two cell-based assays performed at two independent laboratories. MOG-IgG was detected in one patient at a titer of 1:1280 [19]. There were no significant differences between the ION and MS-ON patients as to age, gender, or follow-up time. Median follow-up time was 30 months (range 21–41) for ION, and 28 months (range 19–38) for MS-ON (Table 1).

**Table 1:** Distribution of age, sex, and follow-up time in the patient groups

	*N*	f/m	Ratio	Years of age, median (range)		Follow-up time, median (range)	
HC	12	5/7	0.7	48.0 (16–63)		n.a.	
ION	24	16/8	2.0	39.3 (16–67)		29.6 (21–41)	
MS-ON	16	11/5	2.2	41.5 (23–52)		28.2 (19–38)	
*p* value versus		ION	MS-ON	ION	MS-ON	ION	MS-ON
HC		0.175	0.250	0.219	0.368	n.a.	n.a.
ION			0.990		0.745		0.120
MS-ON							

*n.a.* not applicable

### Routine CSF biomarkers are increased in MS-ON patients and distinguish MS-ON from ION

**Fig. 1:**
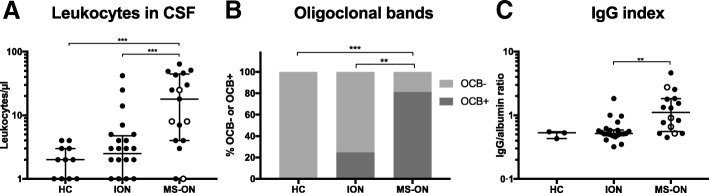
Routine cerebrospinal fluid findings differentiate patients who later converted to multiple sclerosis at onset of optic neuritis. **a** Cerebrospinal fluid (CSF) leukocyte count, **b** distribution of oligoclonal band (OCB) positivity and negativity and **c** IgG index in multiple sclerosis (MS)-related optic neuritis (ON) (MS-ON), isolated ON (ION), and healthy controls (HC). Open circles indicate four patients that were diagnosed with MS 3 to 12 months after ON onset

CSF biomarkers were assessed at the time of the acute ON event. ON patients were stratified based on prospective diagnosis into ION and MS-ON and compared to HC. As a whole, MS-ON patients showed increased levels of CSF leukocytes (median [25th–75th percentiles] 18 [4–44.5] versus 2.5 [1–4.8] cells/μl, respectively, q = 0.0007), they exhibited OCB positivity more frequently than ION patients (13/16 positive versus 6/24, respectively, *q* = 0.0021), and had a higher IgG index (1.1 [0.56–1.82] versus 0.52 [0.49–0.59], respectively, *q =* 0.0052; Fig. 1a–c). Two MS-ON patients but none with ION had albumin ratios above the threshold of [age]/25 + 8 proposed by Hegen and colleagues [15]. The findings suggest higher immune reactivity in the CSF of MS-ON patients.

### High intrathecal IL-10, TNF-α, and CXCL13 are associated with a diagnosis of MS

**Fig. 2:**
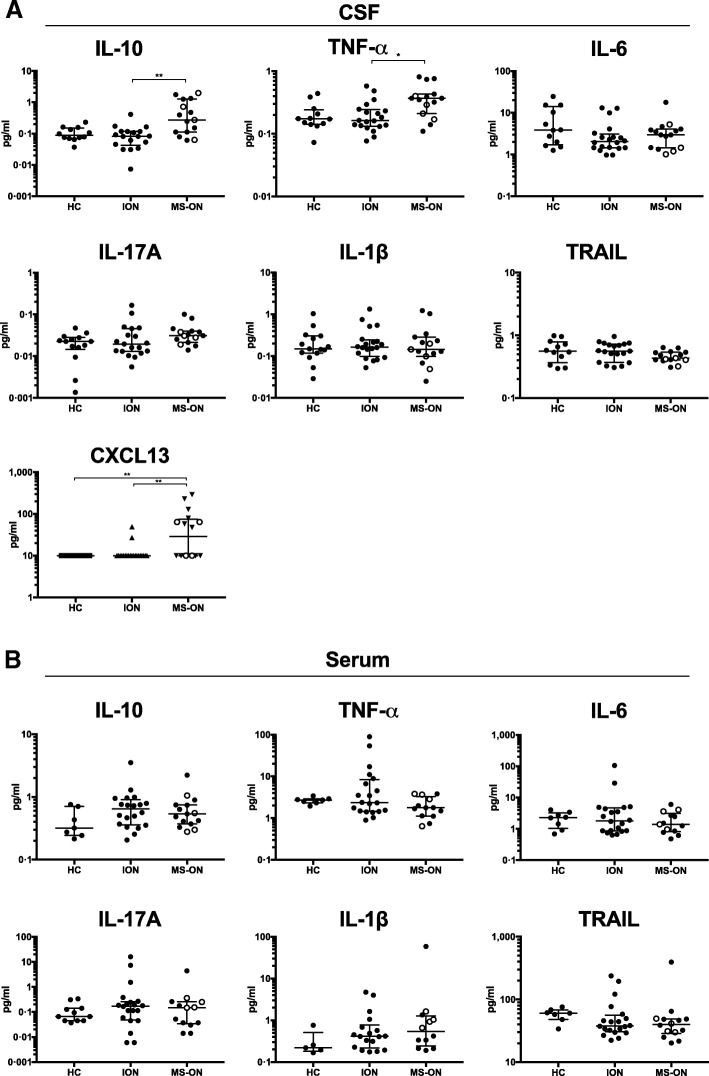
Intrathecal levels of TNF-α, IL-10, and CXCL13 distinguish patients who develop multiple sclerosis. Cytokines and chemokines as denoted in **a** cerebrospinal fluid and **b** serum. Open circles indicate patients that were diagnosed with multiple sclerosis 3 to 12 months after optic neuritis onset

When comparing cytokine/chemokine protein levels, we found higher CSF levels of IL-10, TNF-α, and CXCL13 in MS-ON than in ION (IL-10, respectively, 0.27 [0.11–1.26] vs 0.083 [0.043–0.12] pg/ml, *q =* 0.004, TNF-α 0.37 [0.21–0.43] vs 0.16 [0.13–0.24] pg/ml, *q =* 0.021, and CXCL13 28.9 [10–75.4] vs 10 [10] pg/ml, *q =* 0.0006; Fig. 2a). No differences could be detected in serum (Fig. 2b). This indeed suggests that, although clinically indistinguishable, patients destined to develop MS have a distinct CNS immune phenotype relative to ION patients.

IL-1β, IL-6, IL-17A, and TRAIL could be detected in all CSF samples except IL-1β and IL-17A that were undetectable in two samples, and levels appeared similar between the two patient groups (Fig. 2a, b). IFN-α was detected in serum and CSF from one ION patient while IFN-β could be detected only in CSF from one other ION patient (not shown).

We calculated the cytokine-specific CSF/serum ratios normalized to albumin ratio, e.g., ([IL-10]_CSF_/[IL-10]_serum_)/albumin ratio, to indicate whether cytokines were synthesized intrathecally. Both IL-10 and TNF-α showed significantly higher indices in MS-ON compared to ION before false discovery rate adjustment (*p* = 0.036 and 0.048, respectively). For CXCL13, only one patient with MS-ON had detectable levels in both serum and CSF. The seven other MS-ON patients and two ION patients with detectable CXCL13 in CSF had undetectable levels in serum. These findings argue in favor of intrathecal cytokine synthesis.

**Fig. 3:**
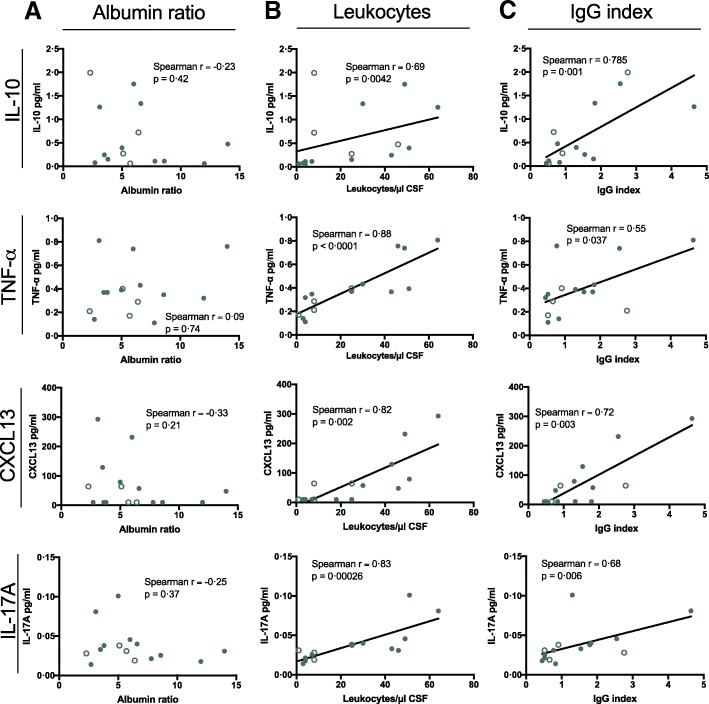
Intrathecal immune activity in patients who develop multiple sclerosis. Cerebrospinal fluid levels of TNF-α, IL-10, IL-17A, and CXCL13 in patients who develop multiple sclerosis (MS-ON) and their correlation with **a** albumin ratio, **b** leukocyte count, and **c** IgG index. Open circles indicate patients that were diagnosed with MS 3 to 12 months after ON onset

Testing for correlations between intrathecal markers for inflammation in MS-ON disclosed increased CSF levels of IL-10, TNF-α, and CXCL13 that failed to correlate with the albumin ratio, suggesting that cytokine levels did not reflect blood/CSF barrier disruption (Fig. 3a). In contrast, a strong correlation between each of these cytokines and CSF leukocyte counts (Spearman *r* ≥ 0.69 for all, *p* = 0.0042, *p* < 0.0001, and *p* = 0.002, respectively; Fig. 3b) and IgG index could be detected (Spearman *r* ≥ 0.55 for all, *p* = 0.001, *p* = 0.037, and *p* = 0.003, respectively; Fig. 3c). IL-17A exhibited a similar pattern of correlation, although not differentially expressed in MS-ON (Fig. 2a).

Similar correlations were also found in ION patients but were less strong (Spearman *r* between 0.48–0.53 and *p* > 0.024 for all; Additional file 1: Figure S1), suggesting that CNS immune processes in MS-ON are distinct from those in ION.

### CSF NF-L levels are increased in acute ON and progress in MS-ON

**Fig. 4:**
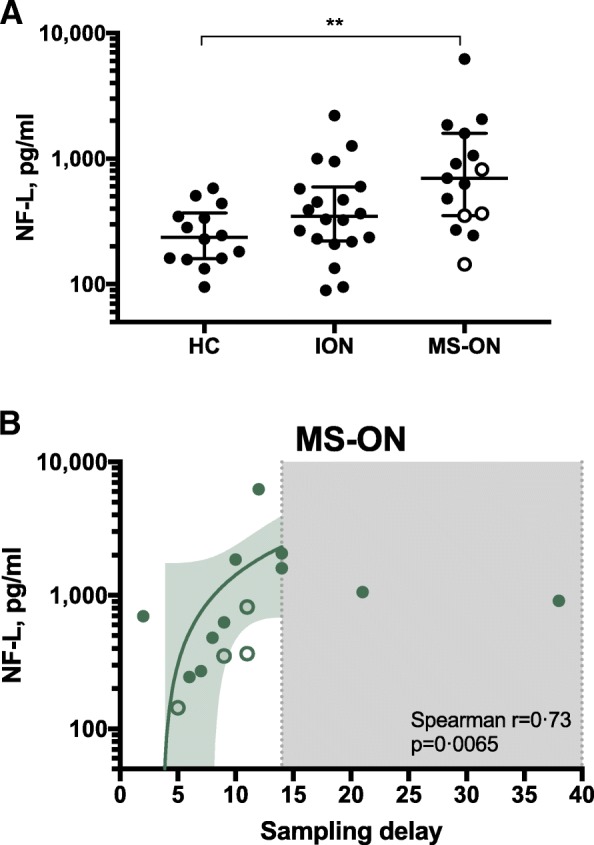
Increased cerebrospinal fluid neurofilament light chain (NF-L) levels in patients who progress to multiple sclerosis. **a** Levels of intrathecal NF-L and **b** significant correlation of NF-L levels in multiple sclerosis (MS)-related optic neuritis (ON) patients with cerebrospinal fluid (CSF) sampling delay from symptom onset. Open circles indicate patients that were diagnosed with multiple sclerosis 3 to 12 months after optic neuritis onset. Excluded from analysis are two samples due to few data points in this time interval

Quantification of NF-L in CSF revealed higher levels in all 40 acute ON patients compared to 11 HC (451 [224–947] pg/ml vs 229 [161–346] pg/ml, respectively, *q =* 0.0324). When stratified based on subsequent (prospective) diagnosis, these elevations in NF-L were restricted to MS-ON patients (*q =* 0.0077, Fig. 4a). Moreover, when considering all ON patients (disregarding the prospective MS-ON or ION diagnosis), patients with CSF NF-L higher than the median (> 450 pg/ml) had more abundant CSF leukocytes than ON patients with NF-L < 450 pg/ml (10.5 cells/μl [3–42.2; *n* = 18] versus 2.0 [1–7.5; *n* = 17], *p* = 0.011, not shown). There was no correlation with other inflammatory markers. In MS-ON, but not in ION patients, a gradual increase in CSF NF-L levels was observed in the period between 7 and 14 days after disease onset (Spearman *r* = 0.73, *p* = 0.0065, Fig. 4b).

### Modeling of conversion of ON to MS

To assess whether it is possible to predict development of MS-ON in the cohort of patients with ON, we employed logistic regression utilizing one or more possible predictor variables. Regression models based on individual parameters were tested first, and optimal cutoffs for each univariate prediction model were calculated according to the Youden method (Additional file 2: Table S1). ROC curves for single predictors are shown in Additional file 3: Figure S2.

**Fig. 5:**
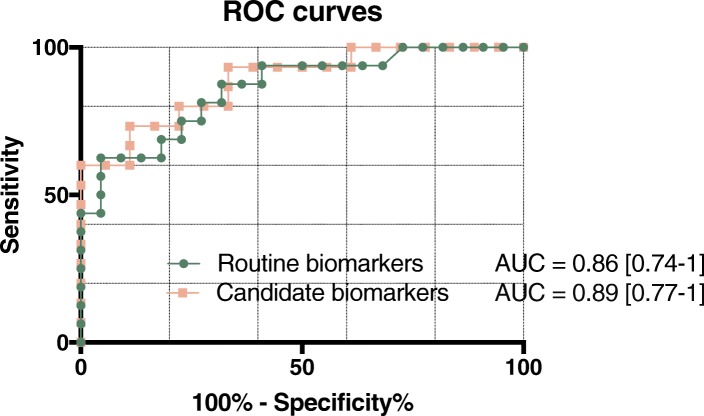
Receiver-operating characteristic (ROC) for models predicting the conversion of optic neuritis to multiple sclerosis. ROC curves showing the discriminative performance of our prediction models. ROC curves for logistic regression models comprising levels of IgG index, cerebrospinal fluid (CSF) leukocytes, and oligoclonal band status, denoted as “routine biomarkers” (green) and CSF IL-10, CXCL13, and NF-L, denoted as “candidate biomarkers” (apricot). Given is area under curve (AUC) and 95% confidence intervals

Next, the predictive performance of two different combined models was tested. The model entitled “routine biomarkers” comprises parameters distinguishing ION from MS-ON in Fig. 1. The second, “candidate biomarkers”, contains parameters based on findings in Figs. 2 and 4. TNF-α was not included because screening sets of variables indicated that it explained the same variation as IL-10 and thus did not add additional information. However, both IgG index and OCB were included in the routine biomarker model even though the ROC curve for leukocytes and OCB showed AUC equivalent to that of leukocytes and IgG index (Additional file 4: Figure S4 and Additional file 5: Figure S5) because of the face validity (i.e., biological relation to MS-ON), thus potentially providing predictive information. Routine biomarkers comprised OCB, leukocyte count, and IgG index, while candidate biomarkers included CSF IL-10, CXCL13, and NF-L. These biomarker cohorts yielded AUC of 0.86 [0.74–0.98] and 0.89 [95% C.I.: 0.77–1], respectively (Table 2 and Fig. 5; cf. Additional file 6: Figure S5 for calibration plots). Combining these two models with six predictors did not significantly improve AUC (0.90; 95% CI: 0.78–1), and thus this aggregate was felt to be too cumbersome for the clinical setting. For all three models, Hosmer-Lemeshow’s goodness-of-fit test supported that the prediction models fitted the data.

**Table 2:** Multivariate logistic regression modeling for the candidate and routine biomarkers and a combined biomarker model, which combines candidate and routine biomarkers into one model

	Number of observations	Likelihood ratio *χ*2	*p* value	Adjusted McFadden *R*^2^	Hosmer-Lemeshow’s *p* value	Area under ROC curve	AUC 95% CI
Experimental biomarkers							
IL-10 NF-L CXCL13	33	17.26	0.0006	0.15	0.66	0.89	0.77–1.00
Routine biomarkers							
OCB Leukocytes IgG index	38	16.33	0.0010	0.16	0.86	0.86	0.74–0.98
Full model	33	21.53	0.0015	0.10	0.66	0.90	0.78–1.00

Because the two predictor models were generated and tested on the same dataset, they are inherently optimistic. Therefore, the optimism of each model was estimated to obtain an optimism-corrected estimate of the AUC. This reduced their AUC to 0.83 (routine) and 0.87 (candidate).

### MS risk assessment via nomograms based on CSF findings

**Fig. 6:**
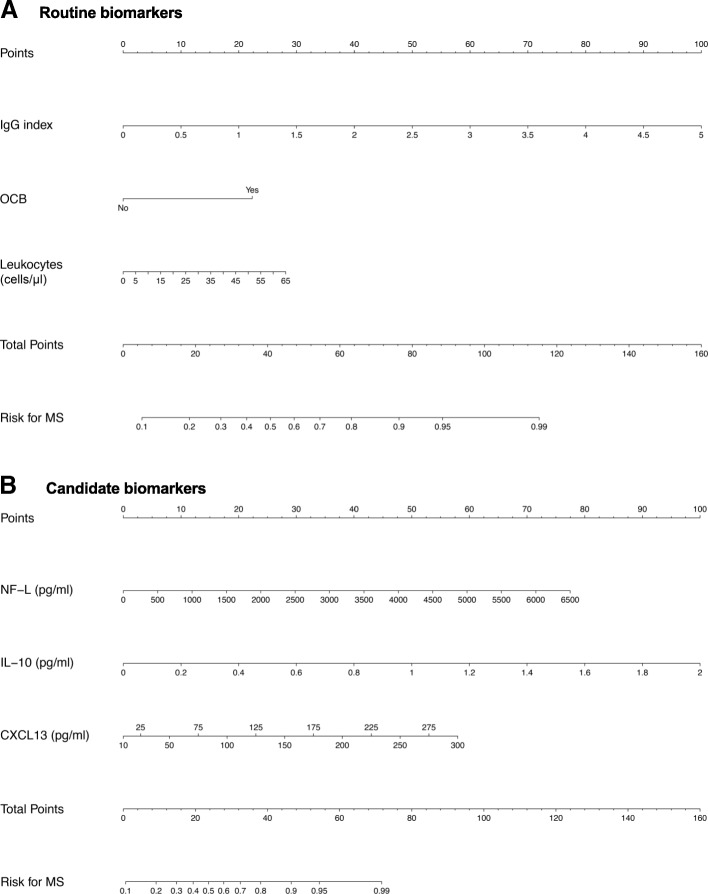
Nomograms for **a** routine and **b** candidate biomarker prediction models for estimation of the patient-specific risk of multiple sclerosis. Each of the nomograms can estimate a patient’s risk of multiple sclerosis (MS) diagnosis. Instructions for use (here exemplified for the “routine model”): Locate the patient’s IgG index value on the “IgG index” scale and find the corresponding point score straight above on the “Points” scale. Do this for the other axes and sum the total point score. Locate the total score on the “Total Points” axis and draw a line straight down on the “Risk for MS” axis: This is the patient’s estimated risk of MS diagnosis within the first year from onset of optic neuritis (ON). Risk of MS should be considered with an uncertainty of up to ± 10% for the “routine biomarker” model, and ± 15% for the “candidate biomarker” model according to calibration plots (Additional file 3: Figure S3). Example of patient from this study: **a** A patient had no OCBs, 3 leukocytes/μl, and an IgG index of 0.48, giving a score on the “routine biomarker” nomogram of 0, 1, and 9 points, respectively (10 in total). This corresponds to approximately 13% risk of developing MS. **b** This same patient had CSF measurements of 0.16 pg/ml IL-10, ≤ 10 pg/ml CXCL13, and 229 pg/ml NF-L. This scored 6, 0, and 2 points, respectively, and 18 in total on the “candidate biomarker” nomogram. Such a score corresponds to circa 19% risk of MS. This patient indeed had ION by the end of the follow-up period

Two nomograms were developed as predictive tools based on levels of CSF biomarkers (routine and candidate models) useful for the estimation of a given patient’s risk of developing MS after presenting with acute ON (Fig. 6a, b). For each, three single predictor variables were combined into a multivariate logistic model. This model was finally visualized in a nomogram. Instructions on how to use the nomograms are described in the Appendix.

## Discussion

Intrathecal biomarkers for inflammation and neurodegeneration may become useful for predicting the diagnosis of MS at onset of acute ON, an early inflammatory event. Moreover, they may provide a better understanding of MS pathogenesis. In this prospective population-based cohort study of acute ON with a longitudinal clinical follow-up, we aimed to determine the predictive value of such potential biomarkers. The main finding was that at acute onset of ON and prior to treatment, patients who later receive a diagnosis of MS (i.e., MS-ON) had a significantly different intrathecal inflammatory profile compared to patients who remained as ION. MS-ON patients showed higher levels of CSF leukocytes and higher IgG index, both correlated with CSF IL-10, TNF-α, CXCL13, and IL-17A levels, and these patients more frequently had OCBs.

Moreover, patients developing MS-ON had progressively increasing levels of NF-L, a marker of neuronal damage. These data suggest that dynamic immune processes are evolving in these patients at ON onset. Potentially, these early indicators can be used to predict development of MS. Furthermore, the relatively high predictive power of routine biomarkers (OCB, CSF leukocytes, and IgG index, optimism-corrected ROC area of 0.82) and candidate biomarkers (CSF IL-10, CXCL13, and NF-L, optimism-corrected ROC area of 0.87) suggests these combinations may differentiate MS-ON from ION. To provide a diagnostic tool, nomograms for estimation of a patient’s specific risk of MS diagnosis based on the CSF routine or candidate biomarkers were developed. Such calculations suggest a potential usefulness of composite indices to advance diagnosis, which could be implemented in clinical practice. The potential for identifying those patients who later develop MS would facilitate timely treatment and thus prevent or minimize disability and other consequences of delayed diagnosis.

CSF examination is invaluable in the diagnostic work-up of patients with suspected MS. The recent 2017 MS criteria include the presence of CSF-restricted OCBs in patients presenting with a clinically isolated syndrome (CIS) together with MRI lesions disseminated in space [7, 20]. CSF-restricted OCBs substitute for the requirement of additional symptoms (a second clinical event) or lesions disseminated in time to confirm the diagnosis of MS, based on evidence that these CSF-restricted OCBs are an independent risk factor for the development of MS [20, 21]. CSF examination may identify new biomarkers that predict MS at an early clinical presentation. In the current study, we evaluated CSF cytokine/chemokine levels and routine biomarkers in the acute phase of ON before treatment with corticosteroids. Our findings are congruent with the recent attention directed toward importance of CSF examination, including assessment of the leukocyte numbers and IgG index. We did not find additional predictive value of an abnormal CSF leukocyte count and IgG index. However, in the absence of OCBs, which in general occurs in a limited proportion of cases, IgG index may be of value as a parameter for intrathecal antibody synthesis [7]. Besides their clinical implications, the findings here are of biological relevance since they suggest areas for further interrogation into disease mechanisms. Elevated CSF levels of CXCL13 in MS-ON compared to ION and controls, together with OCB positivity and IgG index, strongly suggest B cell activity in the CNS [22]. Increased IL-10, an important anti-inflammatory and immunosuppressive cytokine, plays an important role in many inflammatory diseases [23, 24]. However, it is unclear what specific role IL-10 plays in MS. Some aspects of IL-10 actions are relevant to B cell behavior in MS, but its involvement may be through other cell types as well [25, 26]. TNF-α is also upregulated in MS-ON. It possesses pro-inflammatory properties and is dysregulated in many systemic and neurologic autoimmune diseases. In MS, TNF-α is upregulated in active brain lesions and in CSF [27]. Although TNF-α is generally considered pro-inflammatory and thus intuitively detrimental in MS, its impact depends on the particular receptor with which it interacts. Two TNF-α receptors (TNFR1 and TNFR2) are expressed by neurons directly and seem to mediate largely opposing effects with respect to direct de- and re-myelination of neurons [27]. Thus, it may represent a double-edged sword in the context of MS due to multiple mechanisms of action.

NF-L levels in CSF are used as an indicator of neuronal damage in the CNS [28]. We found NF-L levels in CSF to be increased in acute ON patients. This finding suggests that damage of neuronal tissues occurs closely to clinical onset [29]. Moreover, NF-L levels significantly increased over time in MS-ON patients between days 7 and 14 after ON onset, suggesting that neuronal damage develops progressively over days or weeks in patients who later develop MS. Collectively, CSF biomarkers reflect immune dysfunction in MS and in this manner predict the development of MS.

Risk evaluation is crucial for the selection of therapy at an early stage of MS. Few studies have evaluated CSF biomarker profiles at the acute onset of ON to attempt prediction of risk for subsequent development of MS. In the current study, we developed two nomograms as predictive tools based on levels of CSF biomarkers (routine or candidate models) which may be useful in estimating this risk (Fig. 6a, b). For each, three single predictor variables were compiled into a multivariate logistic model. This study was based on a small cohort; nonetheless, this cohort was pre-defined from an epidemiological study with a natural history and longitudinal follow-up of patients. The proposed nomograms may benefit from validation in large, multicenter trials.

A notable strength of this study design is the diagnostic algorithm used to prospectively assess a population-based cohort of ON with a high representativity, which has been characterized extensively [14]. Laboratory investigations were performed using largely automated techniques, which strengthen data reliability. To our knowledge, this is the first study quantifying an array of CSF cytokines on the ultra-sensitive Simoa™ platform, which added high resolution in the low-expressing individuals (limit of detection in the range of ~ 0.01–0.002 pg/ml).

## Conclusions

In conclusion, we determined the predictive value of CSF biomarkers for MS development in this prospective population-based study of acute ON. We found a potential for routine as well as candidate model biomarkers to differentiate individuals, who would subsequently develop MS-ON from those who continued to manifest ION during follow-up. These findings suggest that MS-ON patients had strong and highly orchestrated immune processes occurring in the CNS associated with early neuronal damage. We took advantage of both routine and candidate biomarkers to generate two nomograms as tools for predicting a patient’s specific MS risk after onset of acute ON. Additional studies with larger, well-designed cohorts including differential diagnoses and other ethnicities will be needed for validation preferably at multiple centers.

### Additional files

Additional file 1: **Figure S1.** Correlation between intrathecal markers in isolated optic neuritis. This figure depicts the same, albeit much weaker or even absent correlations as shown for patients who later converted to multiple sclerosis (MS-ON) in Fig. 4. (PDF 123 kb)

Additional file 2: **Table S1.** Logistic regression analyses for single predictors. Highlighted in bold are *p* values for which the odds ratio (OR) confidence interval do not overlap the value of 1, as an indicator of association between the highlighted markers and MS-ON. The optimal cutoff value and corresponding sensitivity/specificity is also given. (DOCX 17 kb)

Additional file 3: **Figure S2.** Receiver-operating characteristic (ROC) curves for the six individual predictors that were unified in two models in Fig. 5. (PDF 142 kb)

Additional file 4: **Figure S4. **Receiver-operating characteristic (ROC) for models predicting the conversion of optic neuritis to multiple sclerosis (here for leukocytes and OCBs). Area under ROC curve = 0.8620. (PDF 38 kb)

Additional file 5: **Figure S5.** Receiver-operating characteristic (ROC) for models predicting the conversion of optic neuritis to multiple sclerosis (here for leukocytes and IgG index). Area under ROC curve = 0.8423. (PDF 38 kb)

Additional file 6: **Figure S3.**(A + B) Calibration plots for the prediction models presented as nomograms. The calibration plots assess the agreement between observations and predictions. For a well-calibrated prediction model, if a 10% risk for MS is predicted, the observed frequency of MS should be close to 10% amongst all patients with the same prediction. The apparent calibration curve relies on our original data and a bias-corrected (=optimism-corrected) curve was based on 500 bootstrap samples. (PDF 81 kb)

## Appendix

Instructions on how to use nomograms are provided in the Fig. 6 legend.

As an example, using the “candidate biomarker” nomogram, one patient had 0.40 pg/ml IL-10, so draw a line straight up onto Points axis (top axis) to find the patient’s point. As located on the IL-10 axis, the patient will receive 20 points. Repeat this procedure for the CXCL13 and NF-L: Based on the CXCL13 level of 79.2 pg/ml and NF-L of 698.4 pg/ml, the patient will receive 14 and 8 points, respectively. The reader may then sum the points achieved for each biomarker, which in this case is 20 + 14 + 8 = 42 points, and locate this sum on the Total Points axis (second from the bottom). Draw a line straight down from the Total Points axis to “Risk for MS” scale (bottom) to find the patient’s risk for MS. Here, 42 points correspond to 0.85, so this patient’s estimated risk of MS is 85% (Fig. 6).

The same patient was OCB positive and had 51 leukocytes/μl and IgG index of 1.3. On the routine biomarker nomogram, this would give 22, 22, and 24 points, respectively, and 68 in total on the Total Points axis. This corresponds to circa 83% risk of MS. This patient indeed was diagnosed with MS closely after acute ON.

## Abbreviations

AUC: Area under curve
BBB: Blood-brain barrier
CSF: Cerebrospinal fluid
ELISA: Enzyme-linked immunosorbent assay
HC: Healthy controls
IFN: Interferon
Ig: Immunoglobulin
ION: Isolated optic neuritis
MOG: Myelin oligodendrocyte glycoprotein
MS: Multiple sclerosis
MS-ON: Multiple sclerosis in relation to optic neuritis
NF-L: Neurofilament light chain
NMOSD: Neuromyelitis optica spectrum disorder
OCBs: Oligoclonal bands
ON: Optic neuritis
ROC: Receiver-operating characteristic
TRAIL: TNF-related apoptosis-inducing ligand
