# Supplementary figures and images for: Cerebrospinal fluid biomarkers for predicting development of multiple sclerosis in acute optic neuritis: a population-based prospective cohort study
Source: J Neuroinflammation. 2019 Mar 11;16:59. doi: 10.1186/s12974-019-1440-5 (PMC6410527; doi:10.1186/s12974-019-1440-5)

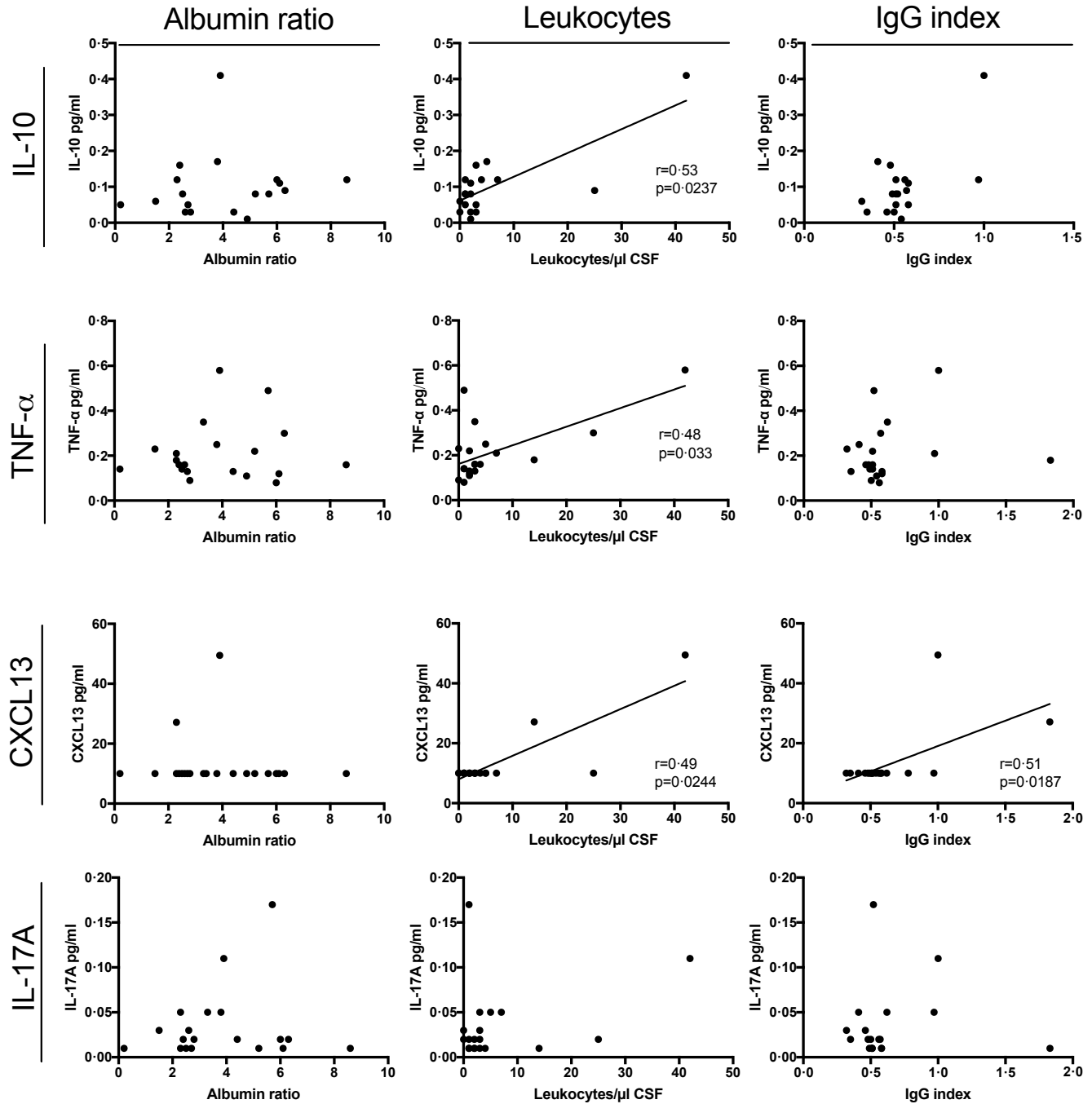

**Supplementary Figure 1: Correlation between intrathecal markers in isolates optic neuritis.**

Supplement: Supplementary file 1 — Figure S1. Correlation between intrathecal markers in isolated optic neuritis. This figure depicts the same, albeit much weaker or even absent correlations as shown for patients who later converted to multiple sclerosis (MS-ON) in Fig. 4. (PDF 123 kb) [file 12974_2019_1440_MOESM1_ESM.pdf]
