# Supplementary material for: Cerebrospinal fluid biomarkers for predicting development of multiple sclerosis in acute optic neuritis: a population-based prospective cohort study
Source: J Neuroinflammation. 2019 Mar 11;16:59. doi: 10.1186/s12974-019-1440-5 (PMC6410527; doi:10.1186/s12974-019-1440-5)
Supplement: Supplementary file 2 — Table S1. Logistic regression analyses for single predictors. Highlighted in bold are p values for which the odds ratio (OR) confidence interval do not overlap the value of 1, as an indicator of association between the highlighted markers and MS-ON. The optimal cutoff value and corresponding sensitivity/specificity is also given. (DOCX 17 kb) [file 12974_2019_1440_MOESM2_ESM.docx]

|  | Maximum likelihood estimates | | | | |  | Odds ratio estimates | | | |  | Applied cutoff | |
| --- | --- | --- | --- | --- | --- | --- | --- | --- | --- | --- | --- | --- | --- |
|  | Log  likelihood | N (obs) | Likelihood ratio χ^2^ | p-value | Adjusted McFadden R^2^ |  | OR | p-value | 95 % confidence  interval | |  | Optimal  cutoff | Sensitivity / Specificity  (%) |
| **CSF** |  |  |  |  |  |  |  |  |  |  |  |  |  |
| TNF-α | -19.71 | 35 | 8.38 | 0.0038 | 0.093 |  | 576 | **0.019** | **2.82** | **>10E5** |  | **0.27 pg/ml** | **73 / 80** |
| IL-10 | -16.30 | 33 | 13.21 | 0.0003 | 0.204 |  | 1,942 | **0.049** | **1.03** | **>10E6** |  | **0.21 pg/ml** | **60 / 94** |
| IL-17A | -23.29 | 34 | 0.08 | 0.78 | -0.084 |  | 18.18 | 0.778 | <10E-6 | >10E6 |  | 0.017 pg/ml | 93 / 47 |
| IL-1β | -23.90 | 35 | 0.01 | 0.92 | -0.083 |  | 1.11 | 0.923 | 0.14 | 8.99 |  | 0.20 pg/ml | 47 / 65 |
| IL-6 | -23.86 | 35 | 0.08 | 0.77 | -0.082 |  | 1.03 | 0.772 | 0.86 | 1.22 |  | 2.67 pg/ml | 67 / 75 |
| TRAIL | -21.06 | 34 | 4.55 | 0.033 | 0.011 |  | 0.01 | 0.052 | 3.1Ε-5 | 1.05 |  | 0.66 pg/ml | 100 / 42 |
| CXCL13 | -19.35 | 37 | 11.91 | 0.0006 | 0.156 |  | 1.05 | **0.030** | **1.00** | **1.10** |  | **37 pg/ml** | **50 / 95** |
| NF-L | -21.84 | 35 | 4.12 | 0.042 | 0.002 |  | 1.00 | 0.086 | 1.00 | 1.00 |  | 614 pg/ml | 60 / 80 |
| **Serum** |  |  |  |  |  |  |  |  |  |  |  |  |  |
| TNF-α | -19.29 | 33 | 5.67 | 0.0173 | 0.038 |  | 0.73 | 0.167 | 0.46 | 1.14 |  | 4.18 pg/ml | 100 / 35 |
| IL-10 | -23.77 | 35 | 0.26 | 0.613 | -0.078 |  | 0.74 | 0.627 | 0.22 | 2.47 |  | 0.75 pg/ml | 80 / 40 |
| IL-17A | -21.70 | 33 | 0.86 | 0·.354 | -0.071 |  | 0.87 | 0.455 | 0.59 | 1.26 |  | 0.041 pg/ml | 38 / 85 |
| IL-1β | -21.33 | 33 | 1.59 | 0.208 | -0.055 |  | 1.07 | 0.463 | 0.90 | 1.27 |  | 0.61 pg/ml | 46 / 75 |
| IL-6 | -21.08 | 33 | 2.08 | 0.149 | -0.043 |  | 0.89 | 0.462 | 0.65 | 1.21 |  | 4.22 pg/ml | 92 / 30 |
| TRAIL | -23.03 | 34 | 0.01 | 0.913 | -0.087 |  | 1.00 | 0.912 | 0.99 | 1.01 |  | 46.1 pg/ml | 43 / 70 |
| **Routine  analyses** |  |  |  |  |  |  |  |  |  |  |  |  |  |
| OCB | -20.46 | 40 | 12.92 | 0.0003 | 0.166 |  | 13.00 | **0.001** | **2.74** | **61.79** |  | **n.a.** | **81 / 75** |
| Leukocytes | -20.66 | 40 | 12.53 | 0.0004 | 0.158 |  | 1.09 | **0.007** | **1.02** | **1.156** |  | **6/µl** | **75 / 83** |
| IgG index | -19.80 | 38 | 12.13 | 0.0005 | 0.157 |  | 11.09 | **0.015** | **1.58** | **77.59** |  | **0.64** | **75 / 82** |
| Albumin ratio | -23.17 | 38 | 5.39 | 0.0202 | 0.027 |  | 1.34 | **0.043** | **1.01** | **1.78** |  | **4.95** | **56 / 77** |

Supplementary Table 1: Logistic regression analyses for single predictors. Highlighted in bold are p-values for which the odds ratio (OR) confidence interval do not overlap the value of 1, as an indicator of association between the highlighted markers and MS-ON.
