# Supplementary material for: Cerebrospinal fluid biomarkers for predicting development of multiple sclerosis in acute optic neuritis: a population-based prospective cohort study
Source: J Neuroinflammation. 2019 Mar 11;16:59. doi: 10.1186/s12974-019-1440-5 (PMC6410527; doi:10.1186/s12974-019-1440-5)
Supplement: Supplementary file 3 — Figure S2. Receiver-operating characteristic (ROC) curves for the six individual predictors that were unified in two models in Fig. 5. (PDF 142 kb) [file 12974_2019_1440_MOESM3_ESM.pdf]

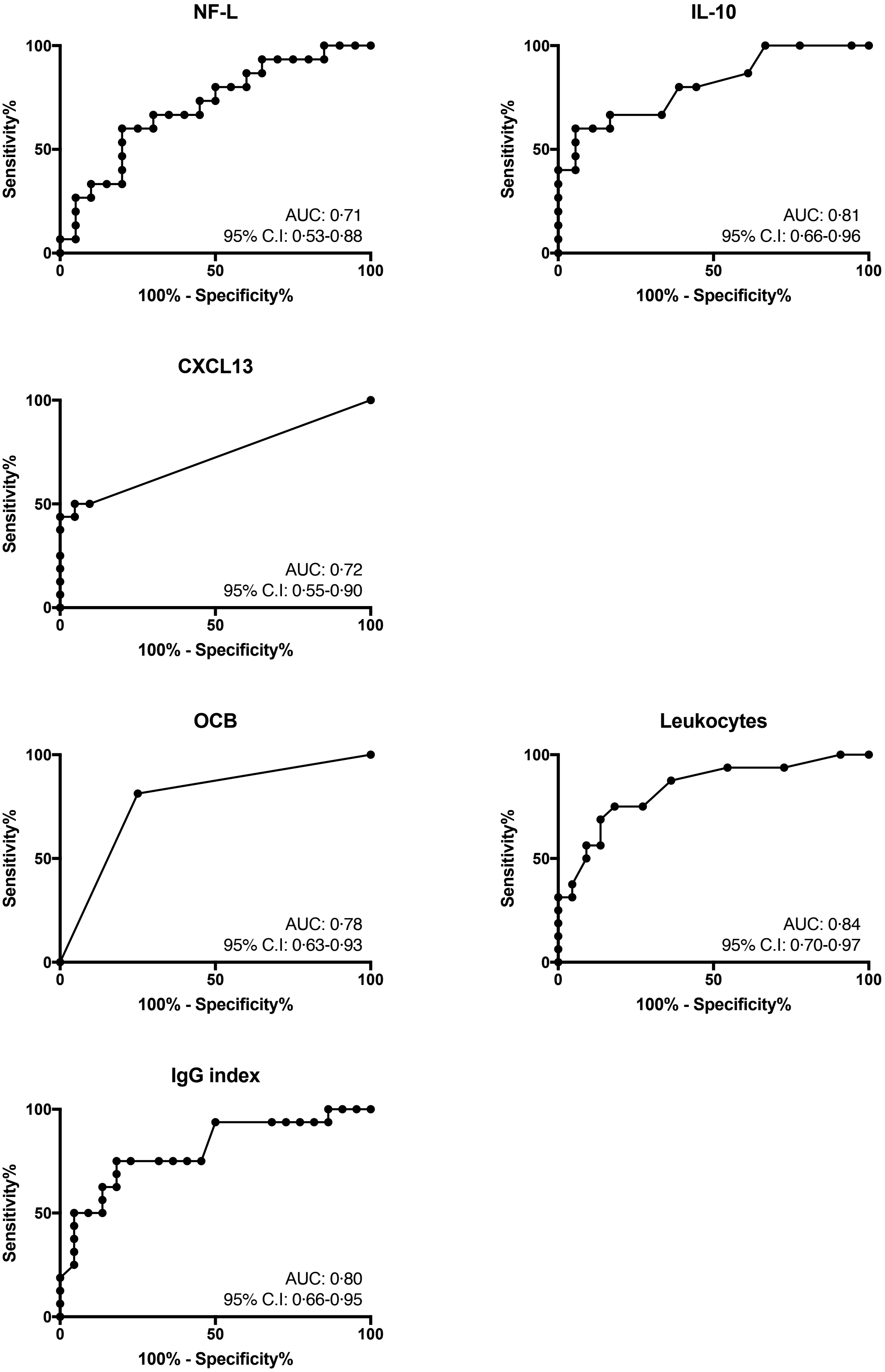

Supplementary Figure 2: Receiver Operating Characteristic curves for the six individual predictors that were unified in two models in Figure 5.
