# Supplementary material for: Cerebrospinal fluid biomarkers for predicting development of multiple sclerosis in acute optic neuritis: a population-based prospective cohort study
Source: J Neuroinflammation. 2019 Mar 11;16:59. doi: 10.1186/s12974-019-1440-5 (PMC6410527; doi:10.1186/s12974-019-1440-5)
Supplement: Supplementary file 5 — Figure S5. Receiver-operating characteristic (ROC) for models predicting the conversion of optic neuritis to multiple sclerosis (here for leukocytes and IgG index). Area under ROC curve = 0.8423. (PDF 38 kb) [file 12974_2019_1440_MOESM5_ESM.pdf]

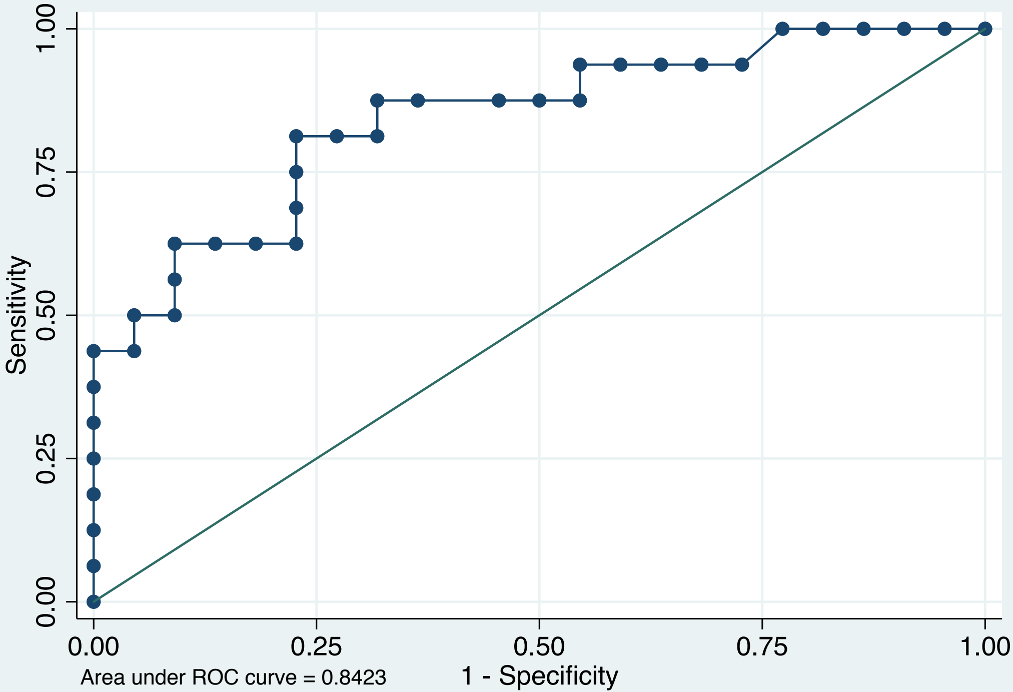

Supplementary Figure 5: Receiver Operating Characteristic (ROC) for models predicting the conversion of optic neuritis to multiple sclerosis (here for leukocytes and IgG index)
