# Supplementary material for: Cerebrospinal fluid biomarkers for predicting development of multiple sclerosis in acute optic neuritis: a population-based prospective cohort study
Source: J Neuroinflammation. 2019 Mar 11;16:59. doi: 10.1186/s12974-019-1440-5 (PMC6410527; doi:10.1186/s12974-019-1440-5)
Supplement: Supplementary file 6 — Figure S3.(A + B) Calibration plots for the prediction models presented as nomograms. The calibration plots assess the agreement between observations and predictions. For a well-calibrated prediction model, if a 10% risk for MS is predicted, the observed frequency of MS should be close to 10% amongst all patients with the same prediction. The apparent calibration curve relies on our original data and a bias-corrected (=optimism-corrected) curve was based on 500 bootstrap samples. (PDF 81 kb) [file 12974_2019_1440_MOESM6_ESM.pdf]

A **Candidate biomarker model**

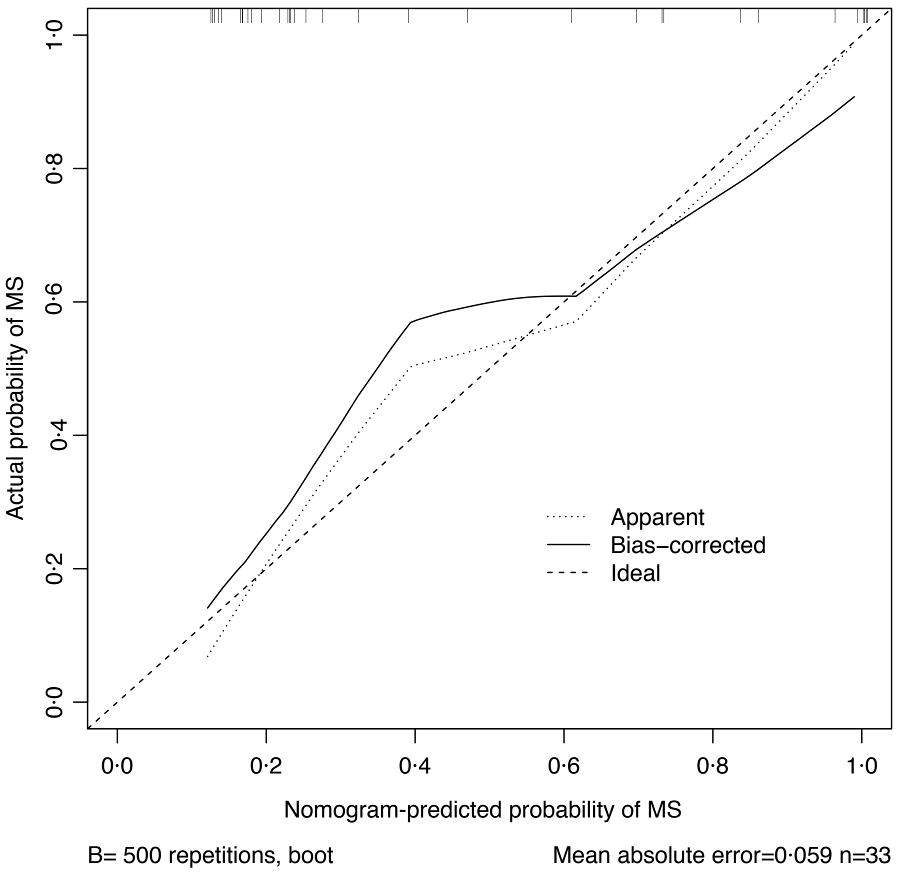

B **Routine biomarker model**

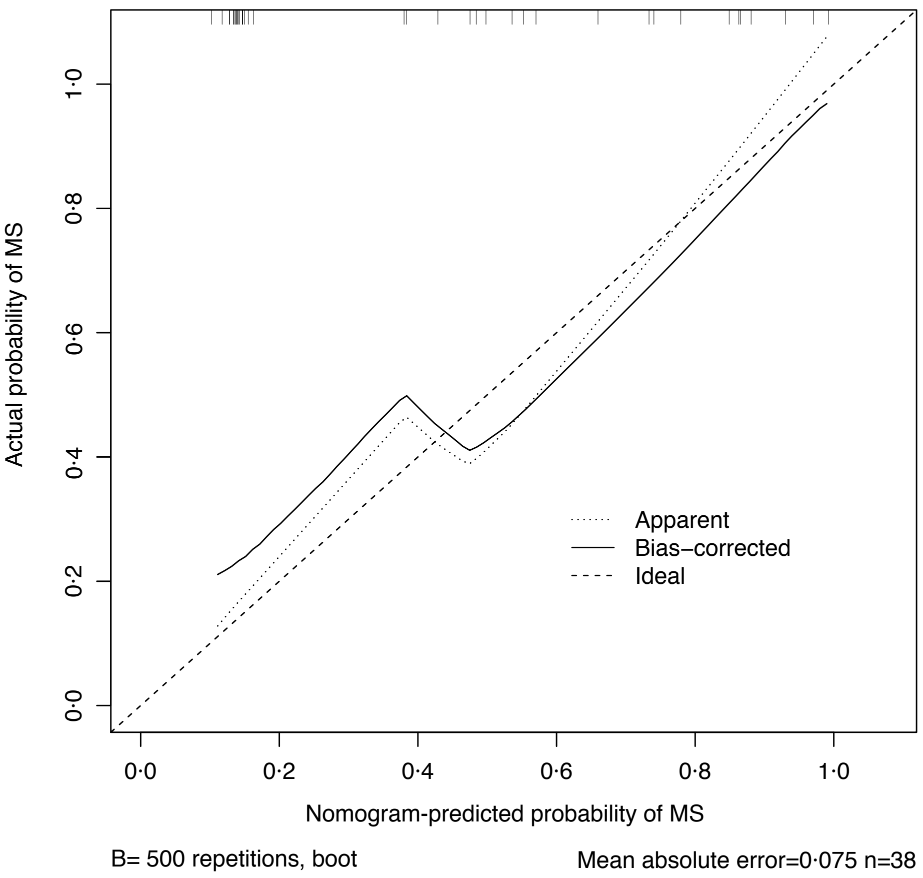

Supplementary Figure 3: Calibration plots for the prediction models presented as nomograms.
